# Blood-biomarkers and devices for atrial fibrillation screening: Lessons learned from the AFRICAT (Atrial Fibrillation Research In CATalonia) study

**DOI:** 10.1371/journal.pone.0273571

**Published:** 2022-08-23

**Authors:** Elena Palà, Alejandro Bustamante, Josep Lluis Clúa-Espuny, Juan Acosta, Felipe González-Loyola, Sara Dos Santos, Domingo Ribas-Segui, Juan Ballesta-Ors, Anna Penalba, Marina Giralt, Iñigo Lechuga-Duran, Delicia Gentille-Lorente, Alonso Pedrote, Miguel Ángel Muñoz, Joan Montaner

**Affiliations:** 1 Neurovascular Research Laboratory, Vall d’Hebron Institute of Research (VHIR)-Universitat Autónoma de Barcelona, Barcelona, Spain; 2 Stroke Unit, Hospital Universitari Germans Trias i Pujol, Badalona, Barcelona, Spain; 3 Equip d’Atenció Primària Tortosa Est, SAP Terres de l’Ebre, Institut Català de la Salut, Tortosa, Spain; 4 Institut d’Investigació en Atenció Primària IDIAP Jordi Gol, Unitat de Suport a la Recerca de Barcelona, Barcelona, Spain; 5 Department of cardiology, Hospital Universitario Virgen del Rocio, Sevilla, Spain; 6 Gerència Atenció Primària de Barcelona, Institut Català de la Salut, Barcelona, Spain; 7 CAP Horta 7F, Àmbit d’Atenció Primària Barcelona ciutat, Institut Català de la Salut, Barcelona, Spain; 8 EAP Sant Pere i Sant Pau, Institut Català de la Salut, Tarragona, Spain; 9 Biochemical department, Hospital Universitari Vall d’Hebron, Barcelona, Spain; 10 Servicio Cardiología, Hospital Virgen De La Cinta, Institut Català Salut Tortosa, Tarragona, Spain; 11 Institute de Biomedicine of Seville, IBiS/Hospital Universitario Virgen del Rocío/CSIC/University of Seville & Department of Neurology, Hospital Universitario Virgen Macarena, Seville, Spain; UNITED STATES

## Abstract

**Background and objective:**

AFRICAT is a prospective cohort study intending to develop an atrial fibrillation (AF) screening program through the combination of blood markers, rhythm detection devices, and long-term monitoring in our community. In particular, we aimed to validate the use of NT-proBNP, and identify new blood biomarkers associated with AF. Also, we aimed to compare AF detection using various wearables and long-term Holter monitoring.

**Methods:**

359 subjects aged 65–75 years with hypertension and diabetes were included in two phases: Phase I (n = 100) and Phase II (n = 259). AF diagnosis was performed by baseline 12-lead ECG, 4 weeks of Holter monitoring (Nuubo^TM^), and/or medical history. An aptamer array including 1310 proteins was measured in the blood of 26 patients. Candidates were selected according to p-value, logFC and biological function to be tested in verification and validation phases. Several screening devices were tested and compared: AliveCor, Watch BP, MyDiagnostick and Fibricheck.

**Results:**

AF was present in 34 subjects (9.47%). The aptamer array revealed 41 proteins with differential expression in AF individuals. TIMP-2 and ST-2 were the most promising candidates in the verification analysis, but none of them was further validated. NT-proBNP (log-transformed) (OR = 1.934; p<0.001) was the only independent biomarker to detect AF in the whole cohort. Compared to an ECG, WatchBP had the highest sensitivity (84.6%) and AUC (0.895 [0.780–1]), while MyDiagnostick showed the highest specificity (97.10%).

**Conclusion:**

The inclusion and monitoring of a cohort of primary care patients for AF detection, together with the testing of biomarkers and screening devices provided useful lessons about AF screening in our community. An AF screening strategy using rhythm detection devices and short monitoring periods among high-risk patients with high NT-proBNP levels could be feasible.

## 1. Introduction

Atrial fibrillation (AF) is one of the most common cardiac arrhythmias in the general population and its incidence and prevalence are increasing globally, becoming an important public health problem [1]. Treatments exist to prevent the most important complications of AF as is the case of oral anticoagulants (OACs) for stroke risk reduction [2]. However, this condition is frequently asymptomatic and paroxysmal, difficulting its diagnosis. As a consequence of the failure to detect AF at early stages, 10% of strokes are caused by previously unknown AF [3]. Screening strategies would potentially increase AF detection rates and reduce its complications, but the best screening approach is not clear [4].

During recent years, a diversity of devices and new methodologies for AF detection have appeared, ranging from single time-point to long-term monitoring devices capable to identify brief asymptomatic AF episodes [5]. Although intensive monitoring periods increase the AF diagnostic yield [6], screening strategies in the primary care setting require inexpensive and cost-effective methodologies.

The incorporation of blood biomarkers into AF screening strategies is gaining interest. It represents an opportunity to enlarge the window for AF detection as some of them might present a kind of “biological memory” being elevated even outside AF episodes. A promising biomarker in this context is N-terminal pro B-type natriuretic peptide (NT-proBNP), which we recently found elevated in AF individuals, even in paroxysmal cases [7]. However, the usefulness of this biomarker needed to be confirmed and potentially complemented by others.

In the present work, we aimed to explore several tools to be applied in an AF screening program in the primary care health setting. In particular, we aimed to validate the use of NT-proBNP, and identify new blood biomarkers associated with AF. Also, we aimed to compare AF detection using various wearables and long-term Holter monitoring.

## 2. Methods

### 2.1. Study population

**Fig 1:**
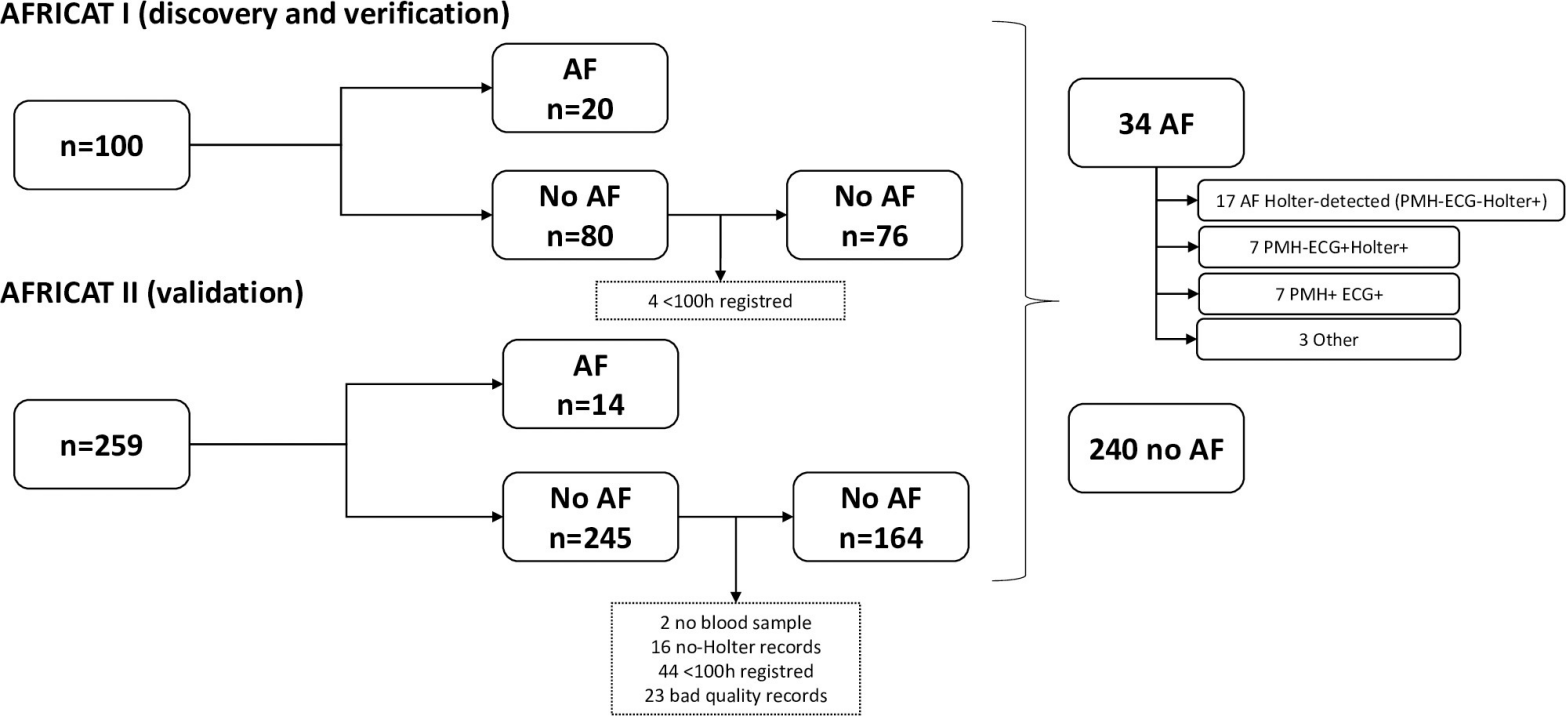
Flow chart of the AFRICAT study.

AFRICAT (Atrial Fibrillation Research in CATalonia; NCT03188484) is a prospective, multicenter, population-based screening study for AF. The study was divided into two phases: Phase I (2016–2017) for discovery and verification, and Phase II (2019–2020) for validation (Fig 1). Individuals aged 65–75 with a registered diagnosis of hypertension and diabetes were identified from the clinical records of three different Catalonia health areas (SAP Muntanya, SAP Reus, and SAP Terres de l’Ebre) and invited to participate in the study. Individuals with chronic inflammatory diseases, active cancer, or dementia were excluded. In Phase II, patients with a previous diagnosis of AF were also excluded. At a baseline visit, patients received a comprehensive assessment consisting of clinical characteristics (demographic factors, vascular risk factors, medications, comorbidities, and vitals), and electrocardiography assessment. At the same baseline visit, several AF detection devices were tested (Watch BP, MyDiagnostick, and AliveCor or Fibricheck), and blood was collected. Moreover, patients received a wearable Holter device (Nuubo^TM^) and were instructed by local trained researchers to wear it for 4 weeks as described previously [7]. AF diagnosis was performed by a baseline 12-lead electrocardiogram (ECG), 4 weeks monitoring with a wearable Holter device (Nuubo^TM^), and/or medical history. On Holter monitoring, AF was defined as irregular R-R intervals without a P wave signal, lasting for more than 60s. Expert cardiologists blinded to clinical characteristics evaluated the anonymized Holter records and ECGs to identify AF episodes. Holter monitoring was optional in the patients with previous diagnosis of AF included in Phase I. More information about the study protocol has already been published [7].

The AFRICAT study protocol was approved by the clinical research ethics committees of IDIAP Jordi Gol (P15/047) and Hospital Universitari Vall d’Hebron [PR (AG) 133–2015]. All participants signed a written informed consent before inclusion. The study protocol conformed to the ethical guidelines of the 1975 Declaration of Helsinki.

### 2.2. Biomarker quantification

Blood was collected into EDTA and serum tubes at the time of inclusion. After centrifugation at 1500 g and 4°C for 15min, plasma and serum aliquots were frozen at −80◦C until biomarker determination.

#### 2.2.1. Discovery study- Somascan

To identify proteins differentially expressed in AF individuals, an experimental design including discovery, verification, and validation phases was followed.

For the discovery study, protein levels in plasma were assessed using the SOMAscan® platform (SomaLogic Inc., Boulder, CO, USA), an aptamer-based proteomic assay measuring 1305 proteins simultaneously [8]. Normalization and calibration procedures were performed by SomaLogic according to their protocol and final data were reported in relative fluorescent units (RFU) [9]. The most promising candidates from this experiment (according to the best p-value, logFC and, plausible pathophysiological role) were verified in the whole Phase I subjects, and subsequently, the ones with nominal p-value <0.1 were selected to be validated in Phase II.

#### 2.2.2. Verification and validation

For the verification and validation studies, selected proteins were quantified using commercial immunoassays: plasma β-endorphin and plasma interleukin-36 alpha (IL-36A) (MyBioSource, San Diego), plasma metalloproteinase inhibitor 2 (TIMP-2), and serum interleukin-1 receptor antagonist protein (ILIRA) (R&D Systems, Minneapolis), plasma coagulation factor IX antigen (FIX) (Diagnostica Stago, Parsippany), plasma bone morphogenic protein 1 (BMP1), serum C-C motif chemokine 3-like 1 (CCL3L1), plasma dermatopontin (DPT) and serum polymeric immunoglobulin receptor (PIGR)(Elabscience, Houston), and serum low-affinity immunoglobulin gamma Fc region receptor II-a (FcgR-IIa)(Cloud-clone, Wuhan). Interleukin-1 receptor-like 1 (ST2/IL1RL1) was measured by ELISA assay in Phase I (R&D Systems, Minneapolis), and by the Ella technology (Proteinsimple) in Phase II. Several samples were tested in both technologies measuring ST-2 to establish their correlation and reproducibility. Plasma NT-proBNP levels were determined by automated immunoassay in a COBAS c8000 (Roche Diagnostics).

All assays were performed blinded to clinical information and according to the manufacturer’s instructions. All samples were tested in duplicate and inter-assay variation was determined by a commercial control (Human Serum, male AB, USA origin from clotted, SIGMA, ref number H16914; Human plasma K2 EDTA, Innovative Research, ref number IPLA-N) tested in duplicate in each plate. Those samples with duplicated values showing a coefficient of variation (CV) higher than 20% were removed. When inter-assay variation was >20%, biomarker levels were standardized. In each separate phase, samples were randomized through the plates according to AF diagnosis to have the same % of cases in each plate. Therefore, standardization was performed, when needed, dividing all the sample’s concentrations by a coefficient calculated as the plate-specific median between the overall plates median. The commercial control sample was used as a reference to standardize the results for the joined analysis of Phase I and Phase II.

### 2.3. Devices

**Fig 2:**
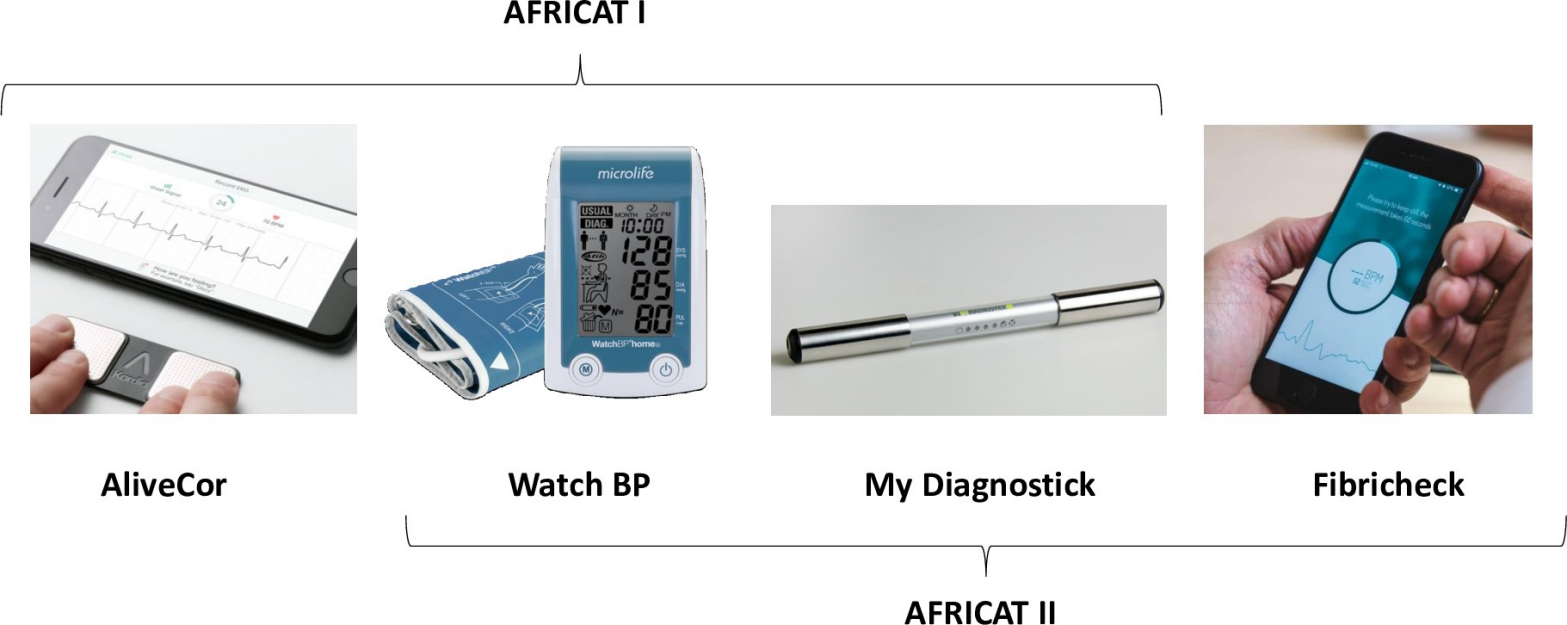
Devices used for AF detection.

At the baseline visit, AF detection devices were tested. At phase I AliveCor, Watch BP, and MyDiagnostick were used. Due to a high rate of unclassified cases (13.3%), AliveCor was replaced by Fibricheck in Phase II (Fig 2).

AliveCor KardiaMobile (AliveCor, USA) [10] is a smartphone-based device that consists of an electrocardiographic recorder of one derivation connected to a mobile phone application.

MyDiagnostick (Applied Biomedical Systems, Netherlands) [11] is a single-lead ECG recorder with the shape of a stick with metallic handles (electrodes) at both ends. After a minute of holding the device with both hands, an indicator (green/red) of possible irregular pulse appears. It operates without additional hardware and can record and store over 100 ECG’s. Microlife WatchBP Home monitor (MicroLife, USA) [12] is an oscillometric digital blood pressure monitor device, which has an algorithm for AF detection based on the regularity of pulses. It automatically takes three sequential measurements to detect possible AF. Fibricheck (Fibricheck, Belgium) [13] is a mobile phone app based on photoplethysmography (PPG) technology. It uses the flashlight and the camera of the mobile phone to measure the changes in blood flow and calculate the heart rate.

For each device, binary information about rhythm (rhythmic vs. arrhythmic) was recorded. Specificity, sensitivity, positive predictive value (PPV), negative predictive value (NPV), and area under the ROC curve (AUC) were calculated for each device in comparison to ECG results. For the analysis, unclassified device records were considered as No AF.

### 2.4. Statistical analysis

Statistical analyses were conducted with SPSS version 20 and R software version 3.6.3. Data were expressed as number (%) for categorical variables and as mean ± SD or median (interquartile range) for continuous variables, depending on its distribution. For univariate analysis, the Mann–Whitney U-test or Student’s t-test was used for continuous variables, and the X^2^ test was used for categorical variables.

The number needed to screen was calculated dividing the number of patients included, by the number of new AF cases detected.

For the discovery experiment, p-values were corrected using false discovery rate (FDR), and base 2 logarithmic fold-changes (logFC) were calculated for each protein by subtracting abundance logarithmic values of the AF to the non-AF samples (logFC  =  log [AF mean/no AF mean]). The Spearman test was used for correlations. Comparisons were first performed between AF patients vs no AF patients, and second between Holter-detected AF vs no AF patients (excluding AF patients diagnosed with other methodologies). Biomarker levels were log-transformed and forward stepwise logistic regression was used to select the ones that independently predicted AF in the whole cohort. Binary logistic regression analyses were performed including the most promising biomarkers and clinical variables of interest (sex, age, heart failure, ischemic cardiopathy, and valvular disease).

The sample size needed for Phase I was calculated aiming to detect a minimum of 10 cases of AF to perform a biomarker case-control discovery experiment and further verification. According to unpublished data from the AFABE study [14], the prevalence of AF in patients with hypertension and diabetes in our community was 9.6%, therefore, a sample of 100 patients was included in phase I. Then, for Phase II sample size was estimated based on phase I biomarker results (power of 80%, α = 0.05) (Ene 3.0, GlaxoSmithKline, UK). According to these calculations, the sample size for Phase II should be between 60, 213, or 282 depending on the biomarker data used for sample size calculation (NT-proBNP, ST-2, or TIMP-2, respectively).

## 3. Results

### 3.1. Patient characteristics and AF detection

100 patients were included in Phase I and 259 in Phase II (Fig 1). The number needed to screen to detect one new AF case in our study was 14.96. Clinical characteristics of the cohort can be found in Table 1.

**Table 1:** Clinical characteristics of the cohort and comparison according to atrial fibrillation diagnosis.

	Phase I AFRICAT n = 100				Phase II AFRICAT n = 259			
	All	AF (n = 20)	No AF (n = 80)^a^	No AF (n = 76)^b^	All	AF (n = 14)	No AF (n = 245)^a^	No AF (n = 164)^b^
**Age**	70 (68–73)	69 (66–71.75)	70 (68–73)*	70 (68–73)	72 (69–74)	71 (69–75)	72 (69–74)*	71 (69–73.75)
**Sex (% female)**	33 (33%)*	7 (35%)	26 (32.5%)*	24 (31.6%)*	153 (59.1%)*	7 (50%)	146 (59.6%)*	93 (56.7%)*
**Alchool**	11 (11%)	1 (5%)	10 (12.5%)	10 (13.2%)	19 (7.4%)	0 (0%)	19 (7.8%)	12 (7.4%)
**Tobacco**	20 (20.2%)*	4 (20%)	16 (20.3%)*	15 (20%)*	23 (8.9%)*	0 (0%)	23 (9.4%)*	17 (10.4%)*
**Dyslipidaemia**	81 (81%)	16 (80%)	65 (81.3%)	61 (80.3%)	212 (81.9%)	10 (71.4%)	202 (82.4%)	133 (81.1%)
**Coronary heart disease**	18 (18%)	8 (40%)*^$^	10 (12.5%)^$^	10 (13.2%)^$^	49 (18.9%)	1 (7.1%)*	48 (19.6%)	33 (20%)
**Heart failure**	3 (3%)	3 (15%)^$^	0 (0%)*^$^	0 (0%)*^$^	23 (8.9%)	0 (0%)	23 (9.4%)*	13 (8%)*
**Valvular disease**	4 (4%)	3 (15%)^$^	1 (1.3%)^$^	1 (1.3%)^$^	12 (4.7%)	1 (7.1%)	11 (4.5%)	8 (4.9%)
**Previous stroke**	6 (6%)	2 (10%)	4 (5%)	2 (2.6%)	18 (6.9%)	0 (0%)	18 (7.3%)	14 (8.5%)
**Anticoagulation**	9 (9%)*	8 (40%)*^$^	1 (1.3%)^$^	1 (1.3%)^$^	2 (0.8%)*	0 (0%)*	2 (0.8%)	1 (0.6%)
**Antiagregation**	50 (50%)	7 (35%)	43 (53.8%)	40 (52.6%)	130 (50.2%)	8 (57.1%)	122 (49.1%)	79 (48.2%)
**SBP**	143.5 (134–153.5)	140.5 (127.5–162.5)	144 (134–151.75)*	144 (134.25–151.75)*	139 (130.5–147.5)	143.5 (130.25–152.25)	138 (130–147)*	136.5 (130–147)*
**DBP**	78 (72.25–86)	79 (73.25–90.75)	78 (71.25–85.75)	78 (71.25–86)	76 (71–83)	74 (70.75–83.75)	76 (71–83)	76 (71.82)

^a^Patients without AF included in the devices analysis

^b^Patients without AF included in the biomarker analysis excluding those without blood samples or short/bad quality registers.

*P-value < 0.05 Phase I vs Phase II comparison

^$^P-value < 0.05 AF vs no AF comparison.

AF, atrial fibrillation; DBP, diastolic blood pressure; SBP, systolic blood pressure.

In general, anticoagulation treatment was more frequent in the AF group (p<0.001). Also, there were some clinical differences between the two phases. In the no AF group, older age (p = 0.015), female sex (p<0.0001), heart failure (p = 0.004), and higher levels of systolic blood pressure (p = 0.004) were more common in Phase II in comparison to Phase I, while smoking (p = 0.010) was more common in Phase I. In the AF group, anticoagulant treatment (p = 0.011) and coronary heart disease (p = 0.005) were more common in Phase I. Median monitoring time was 506 hours (IQR 267h-600h). Holter monitoring was not evaluable in 16 patients, and 44 had short monitoring periods. Therefore, patients with poor Holter records (<100 h registered and/or with artifacts) were excluded from the biomarker analysis to avoid misclassifications (Fig 1). All the patients had a baseline ECG done and therefore were included in the device’s analysis.

**Fig 3:**
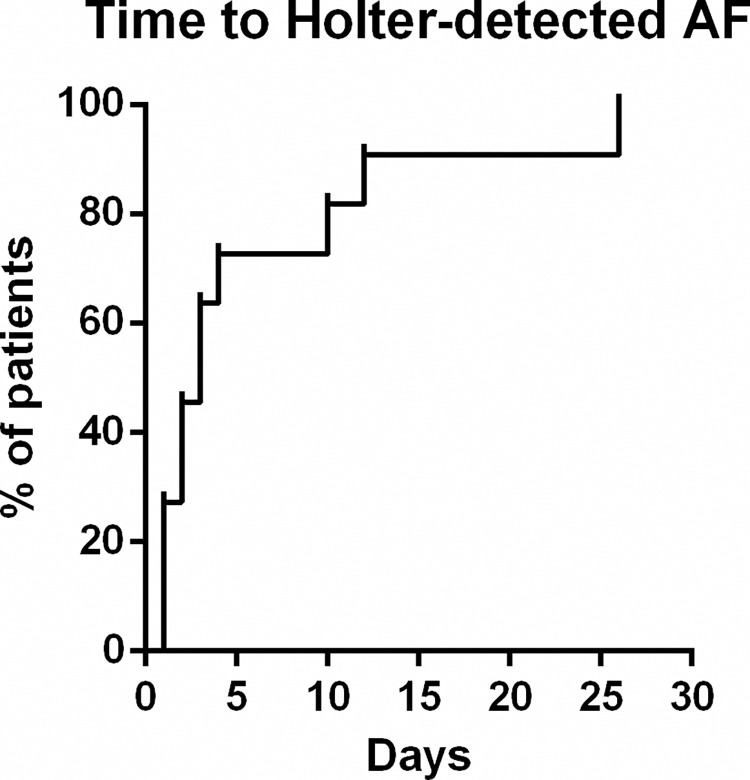
Kaplan-Meier curve reflecting the AF detection time within the patients with Holter-detected AF.

AF was presented in a total of 34 subjects (24 newly detected within the present study). AF cases were classified according to how AF diagnosis was performed in 4 groups, defined by past medical history (PMH) for AF, ECG findings, and Holter AF detection as follows: PMH-ECG-Holter+ (Holter-detected AF), PMH-ECG+Holter+, PMH+ECG+, and others. According to this classification, 17 individuals were only diagnosed during the Holter monitoring period (Holter-detected AF), 7 in Phase I and 10 in Phase II. From these, 82.35% had AF episodes during the first 7 days, and 88.23% during the first two weeks (Fig 3). 7 individuals, 4 in Phase I, and 3 in Phase II, had a new-onset AF on baseline ECG. In all those patients, AF was also found in the Holter register. 7 individuals in Phase I had previous medical history of AF, confirmed by ECG in the baseline visit, and during the Holter monitoring period in those who were followed-up (monitoring in this group was optional). Finally, as others, we classified those patients with an external di-agnosis of AF, previous to the study or during the study, not detected by the ECG or the Holter in the present study.

### 3.2. Biomarker analysis

#### 3.2.1. Discovery

For the discovery study, a subset of 13 AF patients (from which, 7 Holter-detected AF), and 13 no AF patients with long monitoring registers (>450h) were selected from the whole Phase I.

In this subset, patients with AF tended to be younger (p = 0.087) and have higher rates of coronary heart disease (p = 0.073) than no AF patients. Besides, anticoagulation treatment was more common in the AF group (p = 0.039), and antiplatelet treatment was more common in the no AF group (p = 0.006). The other variables were similar between the two groups.

From the evaluated 1305 proteins, 41 proteins had differences between the two groups (nominal p-value<0.05) (S1 Table): 19 had higher levels in patients with AF, and 22 in patients without AF. No protein remained significant after correction by multiple comparisons. 11 candidates were chosen because of the best p-value, logFC and, plausible pathophysiological role to be tested in the whole Phase I (Table 2). Other candidates, like NT-proBNP, were previously tested in this cohort [7].

**Table 2:** Results from the comparisons of biomarker levels between AF and no AF in each phase of the biomarker study.

Name	Uniprot	Discovery (n = 26)			Phase I Verification (n = 96)	Phase II Validation (n = 178)
		p value	FDR	Fold change	P-value	P-value
N-terminal pro-BNP*	P16860	0.001	0.986	2.487	<0.001	0.102
**Dermatopontin (DPT)**	Q07507	0.008	0.986	-0.568	0.758	-
**Interleukin-1 receptor-like 1 (ST-2)**	Q01638	0.015	0.986	0.880	0.064^$^	0.123
**Coagulation factor IX (FIX)**	P00740	0.015	0.986	-0.292	0.015	-
**Metalloproteinase inhibitor 2 (TIMP-2)**	P16035	0.019	0.986	0.245	0.097^$^	0.823
**Beta-endorphin**	P01189	0.026	0.986	-0.419	0.628	-
**Interleukin-1 receptor antagonist protein (IL1RA)**	P18510	0.026	0.986	-0.447	0.311	-
**C-C motif chemokine 3-like 1(CC3L1)**	P16619	0.027	0.986	0.253	0.262^$^	-
**Interleukin-36 alpha (IL36-A)**	Q9UHA7	0.031	0.986	0.491	0.207^$^	-
**Polymeric immunoglobulin receptor (PIGR)**	P01833	0.033	0.986	0.579	0.242^$^	-
**Low affinity immunoglobulin gamma Fc region receptor II-a (FcgR-IIa)**	P12318	0.038	0.986	1.141	0.111^$^	-
**Bone morphogenetic protein 1 (BMP1)**	P13497	0.039	0.986	-0.287	0.937	-

*NT-proBNP was already tested in Phase I as part of another study [7].

#### 3.2.2. Verification

From the 11 proteins tested, TIMP-2 [116.33 ± 23.11 ng/ml vs 106.36 ± 23.67 ng/ml, p = 0.097] and ST-2 [32695.13 pg/ml (IQR 23739.93–44271.92) vs 27488.75 pg/ml (IQR 20538.90–34175.25), p = 0.064] tended to have higher levels in AF patients, and factor IX [118.50±28.01% vs 133.25±22.62, p = 0.015] had lower levels in AF patients (Table 2). The remaining biomarkers were no different between the two groups. None of the biomarkers had significant differences when comparing Holter-detected AF vs no AF.

The known effect of anticoagulation in the factor IX levels [15,16] could not be eliminated from our data, as 40% of the AF patients were previously anticoagulated (mainly due to previous AF history) in comparison to only 1.3% of no AF patients. Therefore, patients on oral anticoagulants were removed from the analysis, and the difference was no longer significant (p = 0.747). For that reason, this protein was not selected to be tested in phase II.

**Fig 4:**
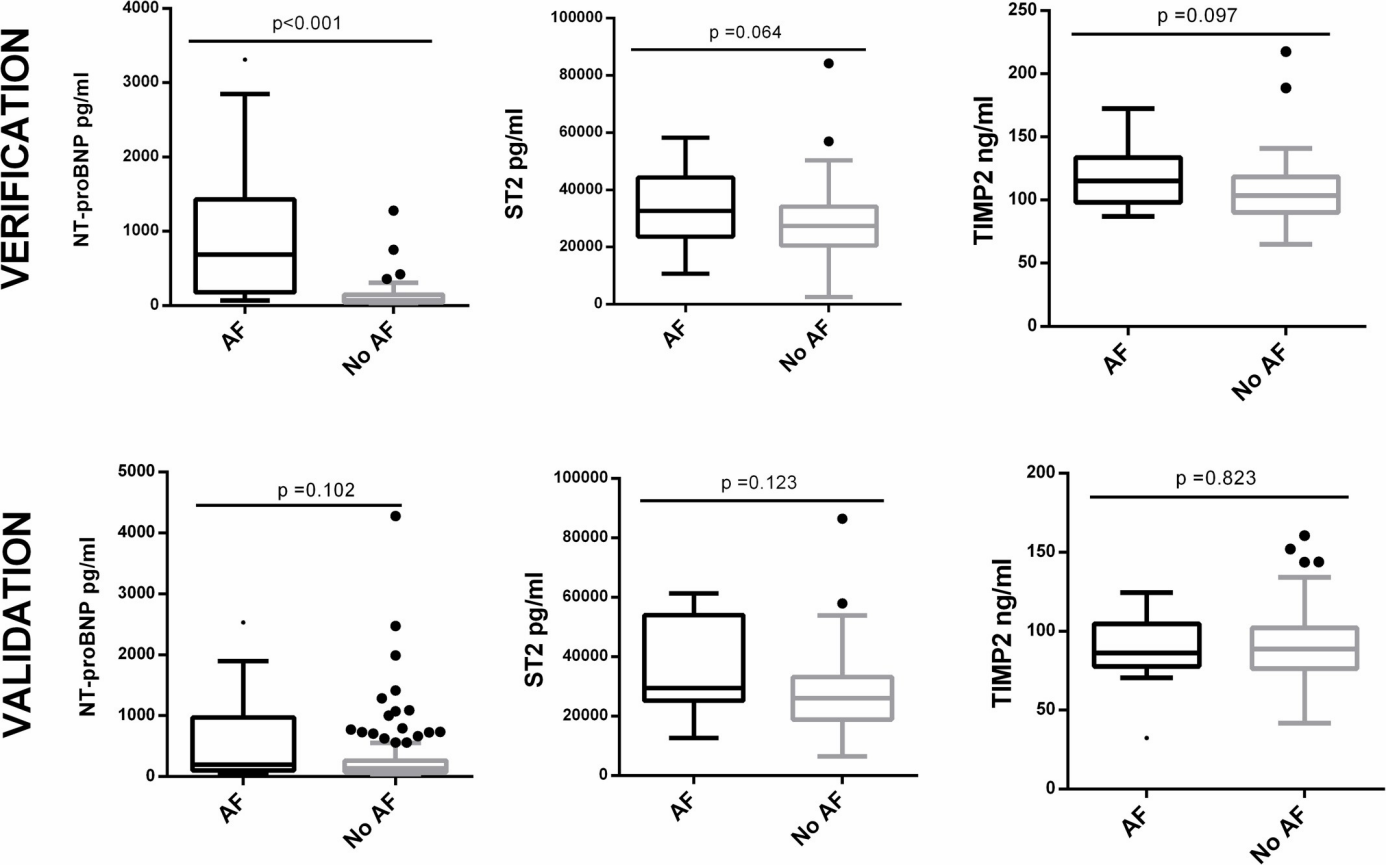
Boxplot distribution of the 3 proteins selected for valiadation (NT-proBNP, ST-2 and TIMP-2) according to AF diagnosis.

Therefore, as shown the best results in this phase, TIMP-2 and ST-2 were selected to be tested in phase II, together with NT-proBNP, which had previously shown promising results, even to detect paroxysmal AF [7] (Figs 4 and S1).

#### 3.2.3. Validation

An automated immunoassay (Ella technology, Proteinsample) was used to test ST-2 in the validation phase. First, 24 samples from the verification phase were also tested with Ella technology to establish the reproducibility between this technique and the one previously used (Quantikine, R&D Systems). Both techniques showed a strong correlation (r = 0.893, p<0.001). TIMP-2 and NT-proBNP were measured with the same techniques as the previous phase.

From the 3 proteins tested, none of them showed significant differences in Phase II (Table 2). Yet, NT-proBNP and ST-2 tended to have increased levels in AF. None of them showed significantly higher concentrations in Holter-detected AF (Figs 4 and S1).

NT-proBNP (log-transformed) (OR = 1.934; 95% CI, 1.525–2.454; p<0.001) was the only biomarker that entered a logistic regression analysis performed in the whole cohort to detect any AF. As a result, the discriminating ability (area under the ROC curve) of NT-proBNP in the whole cohort was 0.771 (95% CI, 0.686–0.856, p<0.0001). If we correct the logistic regression model by forcing the introduction of some clinical variables (sex, age, heart failure, ischemic cardiopathy, and valvular disease), NT-proBNP (OR = 2.101; 95% CI, 1.602–2.755; p<0.001) remained an independent predictor and the predictive ability of the model improved (AUC = 0.807; 95% CI, 0.731–0.882).

The previous suggested cut-off point of NT-proBNP>95pg/ml [7] showed 91.2% sensitivity, 44.2% specificity, 18.8% positive predictive value (PPV), and 97.2% negative predictive value (NPV) to detect any AF and 82.4% sensitivity, 44.2% specificity, 9.5% PPV, and 97.2% NPV to detect paroxysmal AF. Alternatively, the cut-off point of NT-proBNP>125pg/ml showed 76.5% sensitivity, 53.8% specificity, 19% PPV, and 94.2% NPV to detect any AF and 52.9% sensitivity, 53.8% specificity, 7.5% PPV, and 94.2% NPV to detect paroxysmal AF.

### 3.3. Devices

From the 359 included individuals, AF was observed in 14 cases in ECG. MyDiagnostick and WatchBP were used in all the patients. AliveCor was used in the 100 individuals included in Phase I (11 with positive ECG). 13.3% of the results were not interpretable by AliveCor and assigned to No AF. Consequently, this device was substituted by Fibricheck in Phase II, which was used in the 259 remaining individuals (3 with positive ECG). The Fibricheck readings resulted in a low signal in 5 cases (1.93% of results) and were also assigned to No AF.

From the included 359 individuals, one patient was excluded from the comparisons because of the impossibility to use the devices due to left-hand amputation. Also, one patient referred pain during arterial pressure measurement due to an arteriovenous fistula that avoided Watch BP measurement. Table 3 shows the sensitivity, specificity, PPV, PNV, and AUC for each device in comparison to the conventional ECG. Although some devices were only tested in a subset of patients, WatchBP was found as the device showing the best sensitivity (84.6%) and AUC (0.895 [0.780–1]) compared to ECG, while MyDiagnostick showed the best specificity (97.10%). Also, it should be taken into account that some patients with positive results in the previous devices had AF detected during the monitoring period (Table 3).

**Table 3:** Number of AF cases detected by each device and diagnostic performance measures in comparison to ECG.

	AF (All)	AF (ECG)	Sens.	Spec.	PPV	NPV	AUC (ECG ref.) (IC 95%)
**MyDiagnostick (n = 358)**	13	10	76.9%	97.10%	50%	99.1%	0.870 (0.734–1.00)
**WatchBP** **(n = 357)**	15	11	84.6%	94.5%	36.7%	99.4%	0.895 (0.780–1.00)
**AliveCor** **(n = 99)**	10	8	80%	95.5%	66.7%	97.7%	0.878 (0.729–1.00)
**Fibricheck** **(n = 259)**	6	1	33.3%	93.4%	5.6%	99.17%	0.633 (0.267–1.00)

NPV: Negative predictive value; PPV: Positive predictive value. The first column indicates the number of AF from the total detected by each device (here we count AF cases detected by Holter monitoring). The second column indicates the number of AF from the ones detected by ECG, also detected by each device. Sensitivity, specificity, PPV, NPV and AUC were calculated using the ECG as a reference.

## 4. Discussion

In the present study, we aimed to explore several tools to detect AF with the final purpose to develop an AF screening strategy that could be applied in our community. Specifically, we aimed to identify blood biomarkers associated with AF, and compare AF detection using various screening devices and long-term monitoring. As key findings, we validated NT-proBNP to identify patients with AF, but failed to found new biomarkers that could improve its performance. Also, from all the devices tested, we found that WatchBP had the best sensitivity compared to ECG as gold-standard, while MyDiagnostick had the best specificity.

These results were obtained from a cohort of 359 high-risk patients, of which 34 patients had AF. Interestingly, 24 subjects were diagnosed for the first time during the study period and would potentially benefit from anticoagulant treatment to prevent future strokes. A future follow-up of this cohort of patients will provide more data in this regard. It should be taken into account that AF prevalence in phase I was much higher than in phase II, mainly due to the inclusion of already diagnosed AF patients. These patients were included in Phase I to increase the statistical power of the biomarker discovery study. However, to avoid potential selection bias and confounding effects during the biomarker validation, those patients were excluded from phase II.

The number needed to screen to detect one new AF case was less than 15 in our study, much lower than single-time-point screening studies [17]. This is not surprising as diagnostic yield increases with duration, number, and temporal dispersion of the screening [6]. Yet, large monitoring periods are not feasible in screening studies, at least not in every single patient. In our study, patients were mainly diagnosed during the first monitoring days and therefore, we can argue that shorter monitoring periods, for example during one week (capturing 80% of the cases) or during 2 weeks (capturing almost 90% of the cases), would be a good option in this setting. In fact, in the screening context, it has been difficult to convince patients to wear a Holter device for many days. Although few adverse events were reported during the use of the device, some patients complained regarding its discomfort, especially in the summer period, and this was one of the main reasons for denying participation in the study. In the study of Zöga et al [6], when targeting participants with risk factors (older, male, and with higher NT-proBNP) the diagnostic yield of all methodologies increased [6]. Therefore, prioritizing the identification of high-risk individuals to follow for short monitoring periods, even repeated over time, would probably increase patient’s adherence and satisfaction, and decrease costs and workload, while identifying a high proportion of AF cases.

In our study we have already targeted a high-risk population, focusing on hypertensive and diabetic patients of advanced age, conditions that increase the AF prevalence [18]. However, the application of a clinical risk model in this group of patients will optimize even more the selection of candidates to screen. Results have been previously published on the possible relationship between the clinical profile and the risk of AF [19].

As stated before, we confirmed the usefulness of NT-proBNP to detect AF patients in our cohort, but its use was limited in identifying paroxysmal AF cases. Moreover, specificity and positive predictive values were low when aiming a sensitive cut-off (e.g 95 pg/ml or 125 pg/ml). NT-proBNP >125pg/ml was used as a selection tool in the STROKESTOP II study [20] showing higher sensitivity and similar specificity to diagnose AF. However, in their study, as the low-risk group was only screened with an ECG recording the biomarker sensitivity to detect paroxysmal AF could not be tested. Although NT-proBNP is a good candidate, new biomarkers that could complement and improve its predictive accuracy are needed. With this aim, we performed a comprehensive assessment of blood biomarkers by proteomic arrays. However, we should highlight the limitations and difficulties of “discovery” designs in a complex pathology as AF, in which the broad categories under which we classify and compare the patients are not clearly defined. First, we cannot discard the misclassification of subjects in the no AF group. Following the data of Zöga et al [6], 30 days of monitoring would only have identified 34% of patients and some of our records were even shorter. Second, the natural history of AF and the hypothesis that AF may be just a bystander of an underlying disease process called atrial cardiomyopathy that confers stroke susceptibility should be taken into account [21,22]. Therefore, some individuals without AF may have an “AF substrate” with equal stroke risk and be biologically similar to the AF group. All together, these facts add background noise in discovery experiments, especially when performed in a small number of samples. These may be some of the reasons why we have obtained poor results in verifying the discovery study. Another reason may be the inclusion of permanent AF cases in that discovery. This may have revealed proteins increased in permanent AF but not in paroxysmal AF, which are the most interesting cases to identify with biomarkers due to the difficulty to be detected by other methodologies. From all the biomarkers of the discovery experiment, ST-2 and TIMP-2 were selected as the most promising biomarkers. ST2 is a receptor for interleukin-33 and its soluble form, which is released from the myocardium and vascular endothelial cells in response to pressure or volume overload, had been proposed as a promising biomarker for heart failure [23]. It had also been associated with AF, especially regarding its progression [24]. TIMP-2 is an inhibitor of matrix metalloproteinases and its circulating levels in AF patients in comparison to sinus rhythm are controversial [25,26]. Nevertheless, none of these biomarkers were validated in Phase II of the study, probably due to the higher amount of paroxysmal AF cases, which these biomarkers were not able to detect. From both biomarkers, ST-2 showed a trend to be elevated in AF patients and seemed an interesting biomarker but it did not improve the performance of NT-proBNP. Previous discovery experiments have described other biomarkers related to incident or prevalent AF but their use in screening protocols needs to be further assessed [27–29].

Regarding the AF detection devices, in our study, we tested the performance of four devices (MyDiagnostick, WatchBP, AliveCor, and Fibricheck) using different technologies: oscillometry, photoplethysmography, and handled-ECG. It should be noted that the comparison may have been affected by the fact that we echanged one of the devices from Phase I to Phase II, and, therefore, the sample used for all the devices was not the same. Particularlly, the difference in the number of AF cases detected in each phase may have influenced the device’s diagnostic performance measures. For example, the use of Fibricheck uniquely during Phase II, with only 3 patients detected by baseline ECG, biased the calculation of its sensitivity. As a result, the no detection of one of these three patients gave a sensitivity of 33%, which is not accurate and makes difficult its comparison. Regarding the other devices, despite a relatively small sample size, our study showed similar sensitivity and specificity rates to previous studies when compared to ECG [5]. In fact, the meta-analysis performed by Taggar et al [30], stated that blood pressure monitors like WatchBP showed the highest values of sensitivity to detect AF while specificity was higher in non-12-lead-ECG like MyDiagnostick and AliveCor. Nevertheless, sensitivity rates observed seemed insufficient for routine use in clinical practice, at least for a single-time screening purpose. However, most of the tested devices were not intended for single-time use, but their best performance may be obtained after several sequential registers at different time points, even by the patient itself after symptoms presentation. According to our experience, WatchBP, with the highest sensitivity, is one of the most advantageous devices in a clinical setting for population screening strategies. Also, NICE (National Institute for Health and Care Excellence) advocates the use of WatchBP for the detection of AF in patients already being monitored for hypertension taking advantage of the dual functionality of the device [31]. On the other hand, the benefits of MyDiagnostick, AliveCor, and Fibricheck might be higher for patients’ self-monitoring. As ECG confirmation is mandatory by guidelines for the diagnosis of AF, handled ECG devices that provide a verifiable ECG trace, like MyDiagnostick and AliveCor, present a clear advantatge [4].

Although the results obtained in the present project are not accurate enough to implement a screening program, the collaboration of a multidisciplinary team integrating primary care physicians, cardiologists, and researchers working together, provided useful lessons to take into account in primary care AF management and future study designs. Taking into account all our results the use of a clinical predictive model in combination with screening devices (e.g WatchBP or MyDiagnostick) and biomarkers (e.g.NT-proBNP) would probably be useful to select high-risk patients to be monitored for AF detection. Then, selected patients could be monitored continuously during short periods with a wearable Holter device (e.g. 7 days). In our experience, primary care physicians could be in charge of the monitoring with the collaboration of cardiologists to interpret the records. Another option would be the patient self-monitoring using devices or mobile applications (e.g. Fibricheck, AliveCor, MyDiagnostick). The cost-effectiveness of a screening design like that needs to be further evaluated.

Apart from the limitations discussed until now, the main limitation of our study was the reduced sample size. Although the final sample size was inside the sample calculation window, we aimed to a larger sample size in Phase II, corresponding to the upper limit of sample size calculation (n = 282). However, the initiation of the Covid-19 pandemic and the finalization of the financial period for the study limited the inclusion. More important, even though a large number of patients were included, the prospective nature of the study limited the number of AF detected. The limited number of AF patients may prevent generalizability of our results and should be further confirmed. The sample size limitation was particularly important in analyses performed in a subset of patients, like the discovery biomarker experiment. Also, due to some technical problems with the wearable Holter devices, some patients had to be excluded from some analyses, reducing, even more, the statistical power. Finally, the participant selection was not randomized and might have suffered selection bias (eg. healthy user bias, or patients with more cardiac pathologies being more prone to partici-pate). However, this cohort represents a population that would participate in an AF screening study.

## 5. Conclusions

The inclusion and monitoring of a cohort of primary care patients for AF detection, together with the testing of biomarkers and screening devices provided useful lessons about AF screening in our community, despite the limited results obtained. An AF screening strategy using rhythm detection devices and short monitoring periods among high-risk patients with high NT-proBNP levels could be feasible.

Moreover, the present results will contribute to the AFFECT-EU initiative in which information from different European screening studies will permit estimating and drawing conclusions on the efficacy of different screening methods and strategies [32].

## Supporting information

S1 Fig Boxplot distribution of the 3 proteins selected for validation (NT-proBNP, ST-2 and TIMP-2) according to the methodology used for AF diagnosis. (TIF)

S1 Table Top table of the differential expressed proteins between AF and no AF. Results from the discovery study. Proteins selected to be verified in the whole Phase 1 are highlighted in grey. Proteins in bold but not highlighted were already tested in the whole phase 1 as part of a previous published work [7].
(DOCX)

S1 Dataset All patient data and biomarker measurements. (XLSX)

S2 Dataset Somascan raw data. (XLSX)
