# Supplementary figures and images for: Blood-biomarkers and devices for atrial fibrillation screening: Lessons learned from the AFRICAT (Atrial Fibrillation Research In CATalonia) study
Source: PLoS One. 2022 Aug 23;17(8):e0273571. doi: 10.1371/journal.pone.0273571 (PMC9398023; doi:10.1371/journal.pone.0273571)

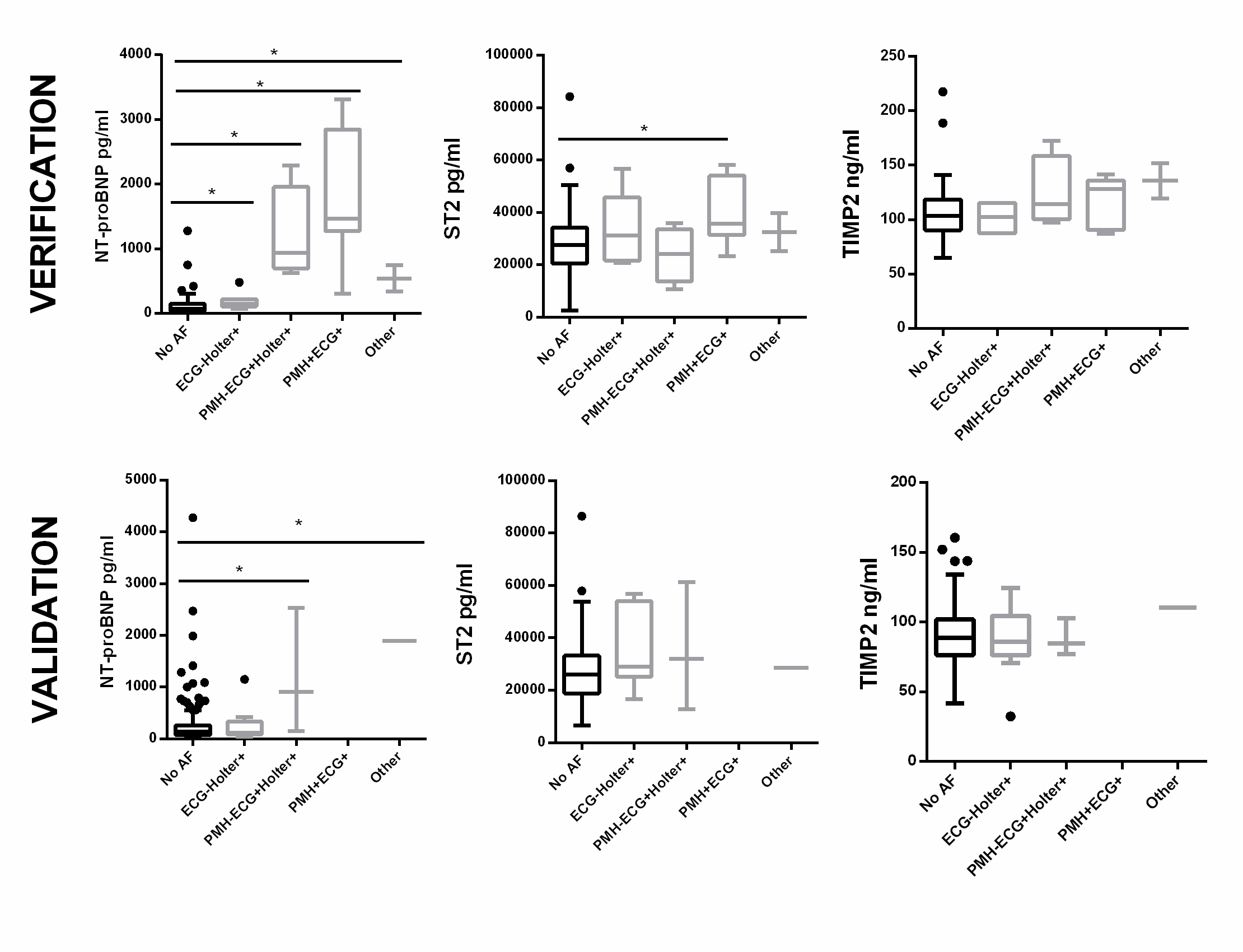

Supplement: S1 Fig — (TIF) [file pone.0273571.s001.tif]
