# Supplementary material for: Blood-biomarkers and devices for atrial fibrillation screening: Lessons learned from the AFRICAT (Atrial Fibrillation Research In CATalonia) study
Source: PLoS One. 2022 Aug 23;17(8):e0273571. doi: 10.1371/journal.pone.0273571 (PMC9398023; doi:10.1371/journal.pone.0273571)
Supplement: S1 Table — Results from the discovery study. Proteins selected to be verified in the whole Phase 1 are highlighted in grey. Proteins in bold but not highlighted were already tested in the whole phase 1 as part of a previous published work [7]. (DOCX) [file pone.0273571.s002.docx]

**S1 Table: Top table of the differential expressed proteins between AF and no AF. Results from the discovery study.**

| Protein name | Uniprot | P.value | FDR | Fold change |
| --- | --- | --- | --- | --- |
| N-terminal pro-BNP | P16860 | 0,001 | 0,986 | 2,487 |
| Dermatopontin (DPT) | Q07507 | 0,008 | 0,986 | -0,569 |
| cAMP-specific 3',5'-cyclic phosphodiesterase 4D | Q08499 | 0,010 | 0,986 | 0,302 |
| Coagulation factor IXab | P00740 | 0,010 | 0,986 | -0,306 |
| Interleukin-1 receptor-like 1 (ST-2) | Q01638 | 0,015 | 0,986 | 0,881 |
| Coagulation factor IX (FIX) | P00740 | 0,015 | 0,986 | -0,292 |
| Low-density lipoprotein receptor-related protein 1, soluble | Q07954 | 0,015 | 0,986 | 0,287 |
| Tumor necrosis factor receptor superfamily member 9 | Q07011 | 0,016 | 0,986 | -0,162 |
| Dual specificity mitogen-activated protein kinase kinase 2 | P36507 | 0,016 | 0,986 | -0,386 |
| Troponin I, cardiac muscle | P19429 | 0,017 | 0,986 | 0,428 |
| Metalloproteinase inhibitor 2 (TIMP-2) | P16035 | 0,019 | 0,986 | 0,244 |
| Thrombin | P00734 | 0,020 | 0,986 | -0,273 |
| 3-hydroxyacyl-CoA dehydrogenase type-2 | Q99714 | 0,020 | 0,986 | 0,358 |
| Chymase | P23946 | 0,022 | 0,986 | 0,187 |
| Brain natriuretic peptide 32 | P16860 | 0,022 | 0,986 | 0,293 |
| Collagen alpha-1(XXIII) chain | Q86Y22 | 0,022 | 0,986 | 0,179 |
| Vitamin K-dependent protein C | P04070 | 0,023 | 0,986 | -0,384 |
| Beta-endorphin | P01189 | 0,026 | 0,986 | -0,419 |
| Laminin | P25391, P07942, P11047 | 0,026 | 0,986 | 0,313 |
| Leucine-rich repeat transmembrane neuronal protein 3 | Q86VH5 | 0,026 | 0,986 | -0,100 |
| Adenylosuccinate lyase | P30566 | 0,026 | 0,986 | 0,013 |
| Interleukin-1 receptor antagonist protein (IL1RA) | P18510 | 0,026 | 0,986 | -0,447 |
| A disintegrin and metalloproteinase with thrombospondin motifs 13 (ADAMTS13) | Q76LX8 | 0,026 | 0,986 | -0,333 |
| C-C motif chemokine 3-like 1 (CCL3L1) | P16619 | 0,027 | 0,986 | 0,253 |
| Transcription factor AP-1 | P05412 | 0,028 | 0,986 | 0,184 |
| Casein kinase II 2-alpha':2-beta heterotetramer | P19784 P67870 | 0,029 | 0,986 | -0,252 |
| Interleukin-36 alpha (IL-36A) | Q9UHA7 | 0,031 | 0,986 | 0,491 |
| C5a anaphylatoxin | P01031 | 0,033 | 0,986 | -0,415 |
| Polymeric immunoglobulin receptor (PIGR) | P01833 | 0,033 | 0,986 | 0,579 |
| Coagulation factor Xa | P00742 | 0,034 | 0,986 | -0,409 |
| Lipopolysaccharide-binding protein | P18428 | 0,036 | 0,986 | 0,165 |
| Histone H2A type 3 | Q7L7L0 | 0,038 | 0,986 | 0,518 |
| Growth hormone receptor | P10912 | 0,038 | 0,986 | -0,503 |
| NKG2D ligand 2 | Q9BZM5 | 0,038 | 0,986 | -0,727 |
| Low affinity immunoglobulin gamma Fc region receptor II-a (FcgR-IIa) | P12318 | 0,038 | 0,986 | 1,141 |
| Fatty acid-binding protein, heart | P05413 | 0,038 | 0,986 | -0,339 |
| Bone morphogenetic protein 1 (BMP1) | P13497 | 0,039 | 0,986 | -0,287 |
| WAP, kazal, immunoglobulin, kunitz and NTR domain-containing protein 1 | Q96NZ8 | 0,043 | 0,986 | -0,375 |
| Fibronectin Fragment 3 | P02751 | 0,043 | 0,986 | -0,340 |
| Mitogen-activated protein kinase 11 | Q15759 | 0,043 | 0,986 | -1,091 |
| Pro-opiomelanocortin | P01189 | 0,048 | 0,986 | -0,740 |

Proteins selected to be verified in the whole Phase 1 are highlighted in grey. Proteins in bold but not highlighted were already tested in the whole phase 1 as part of a previous published work^1^

**References**

1. Palà E, Bustamante A, Clúa-Espuny JL, et al. N-Terminal Pro B-Type Natriuretic Peptide’s Usefulness for Paroxysmal Atrial Fibrillation Detection Among Populations Carrying Cardiovascular Risk Factors. *Front Neurol*. 2019;10(November):1-9. doi:10.3389/fneur.2019.01226
